# Human Non-linguistic Vocal Repertoire: Call Types and Their Meaning

**DOI:** 10.1007/s10919-017-0267-y

**Published:** 2017-09-30

**Authors:** Andrey Anikin, Rasmus Bååth, Tomas Persson

**Affiliations:** Division of Cognitive Science, Department of Philosophy, Lund University, Box 192, 221 00 Lund, Sweden

**Keywords:** Emotion, Non-linguistic vocalizations, Semantic spaces, Cross-linguistic naming study, Triad classification task

## Abstract

Recent research on human nonverbal vocalizations has led to considerable progress in our understanding of vocal communication of emotion. However, in contrast to studies of animal vocalizations, this research has focused mainly on the emotional interpretation of such signals. The repertoire of human nonverbal vocalizations as acoustic types, and the mapping between acoustic and emotional categories, thus remain underexplored. In a cross-linguistic naming task (Experiment 1), verbal categorization of 132 authentic (non-acted) human vocalizations by English-, Swedish- and Russian-speaking participants revealed the same major acoustic types: laugh, cry, scream, moan, and possibly roar and sigh. The association between call type and perceived emotion was systematic but non-redundant: listeners associated every call type with a limited, but in some cases relatively wide, range of emotions. The speed and consistency of naming the call type predicted the speed and consistency of inferring the caller’s emotion, suggesting that acoustic and emotional categorizations are closely related. However, participants preferred to name the call type before naming the emotion. Furthermore, nonverbal categorization of the same stimuli in a triad classification task (Experiment 2) was more compatible with classification by call type than by emotion, indicating the former’s greater perceptual salience. These results suggest that acoustic categorization may precede attribution of emotion, highlighting the need to distinguish between the overt form of nonverbal signals and their interpretation by the perceiver. Both within- and between-call acoustic variation can then be modeled explicitly, bringing research on human nonverbal vocalizations more in line with the work on animal communication.

**Electronic supplementary material:**

The online version of this article (doi:10.1007/s10919-017-0267-y) contains supplementary material, which is available to authorized users.

## Introduction

Emotion is an essential part of being human and a matter of great theoretical and clinical significance. It has justifiably attracted a lot of attention in psychology and neuroscience, including research on facial expressions (Ekman et al. 1969; Izard 1994), prosody (Banse and Scherer 1996), and non-linguistic vocalizations (Belin et al. 2008; Lima et al. 2013). This abiding interest in nonverbal communication has shed light on how affective states can be expressed without words; on the other hand, the most obvious level of analysis, namely the surface form of the signals themselves, has received far less attention.

The relative neglect of alternative, non-affective categories in nonverbal communication may prove a liability, because such categories are both intuitively appealing and useful for research. For example, when researchers analyze the differences between authentic and posed laughter (Bryant and Aktipis 2014; Lavan et al. 2015), evolutionary adaptive value of crying (Provine et al. 2009), or unique acoustic signatures of screaming (Arnal et al. 2015), they implicitly refer to these sounds as acoustic categories that are somehow different from each other and from other sounds. Using the terminology common in animal research, laughter, vocal crying, and screaming are treated as distinct vocalizations, or “call types”. Is this classification justified? In what sense is laughter a call type? What other call types do humans have? A systematic analysis of these issues is the goal of this study.

We begin by justifying the applicability of the concepts and methods of ethology to the study of human non-linguistic vocalizations. We then review the available evidence on the types of vocalizations in human vocal repertoire and present the results of two perceptual experiments that contrast the classification of non-linguistic sounds in terms of emotion and in terms of acoustic categories. Our key objective is to test the hypothesis that acoustic categories (such as a laugh, a scream, etc.) are salient to listeners and not equivalent to affective states.

### Non-linguistic Vocalizations from an Ethological Perspective

Despite some important exceptions (Oller and Griebel 2008; Watson et al. 2015), the acoustic structure of animal vocalizations is largely determined on a genetic level, so that all members of a species produce essentially the same vocalizations (Owren et al. 2011; Wheeler and Fischer 2012). In contrast, human language is not only unusually flexible and powerful as a communicative tool (Devitt and Sterelny 1999), but it is also entirely dependent on a socially transmitted, culture-specific code: We are not born speaking English or Javanese. At the same time, the privileged status of language should not blind us to the fact that many of the sounds humans produce in everyday life are non-linguistic (Provine 2012). There is mounting evidence (reviewed below) that non-linguistic sounds such as laughter are more similar to the vocalizations of other mammals than they are to human language. This evidence comes from neurological research on vocal production and psychological research on vocal perception.

To begin with production, it is well established that the vocal flexibility associated with mastery of language does not preclude the existence of separate, phylogenetically older neural networks responsible for the production of non-linguistic vocalizations (Ackermann et al. 2014; Jürgens 2009). Aphasic patients with lesions in motor cortex (Jürgens 2009) as well as congenitally deaf (Scheiner et al. 2006) and even unencephalic (Newman 2007) infants may laugh and moan just like typical infants. This is possible because non-linguistic vocalizations are controlled by dedicated circuits deep in the brain stem, whereas speech relies on a separate pathway leading from motor cortex directly to laryngeal motoneurons (Jürgens 2009). This separate neural control mechanism explains why it is hard to laugh or cry at will (Provine 2012)—these vocalizations are normally not under direct volitional control. The currently available neurological evidence is not sufficiently detailed to determine precisely how many species-typical vocalizations humans have and what these vocalizations are. However, since the neural machinery controlling vocalizing in mammals is known to be evolutionarily stable (Ackermann et al. 2014), at least some human vocalizations should have direct analogs in the calls of other mammals, and some likely candidates are being investigated (see below).

Moving on to perception, numerous studies have demonstrated that listeners can extract a lot of useful information from sounds that contain little or no phonemic structure. At least eight (Belin et al. 2008; Lima et al. 2013) and perhaps as many as 14–16 (Cordaro et al. 2016; Simon-Thomas et al. 2009) affective states can be correctly identified if a person is instructed to portray them without resorting to language. In addition, listeners can discriminate between authentic and posed emotion (Anikin and Lima 2017; Bryant and Aktipis 2014) or judge whether two people laughing together are friends or strangers (Bryant et al. 2016). Vocalizations of pain (Belin et al. 2008) and physical effort (Anikin and Persson 2017) are also easily recognizable. The information available from non-linguistic vocalizations is thus rich and not strictly limited to emotion.

Recognition accuracy in cross-cultural studies tends to be slightly higher when the speaker belongs to the same group (Elfenbein and Ambady 2002; Koeda et al. 2013; Laukka et al. 2013), demonstrating that even non-linguistic sounds have some culture-specific component. Nevertheless, listeners in even the most isolated communities with little exposure to Western media recognize the emotion expressed by non-linguistic vocalizations of Westerners at above-chance levels (Cordaro et al. 2016; Sauter et al. 2010). The signal system involving laughs and moans is thus much more universal than the expressions of a given language. This raises the question of what the meaningful units of this signal system might be. Are they word-like, as in language?

To answer this question, we need to understand what makes non-linguistic sounds meaningful. A commonly used method is to investigate the communicative significance of particular acoustic features. For example, pitch, intensity, and duration increase with arousal in both human (Scheiner et al. 2002) and animal (Briefer 2012) vocalizations; harsh, noisy sounds are perceived as more aggressive compared to tonal sounds (Anikin and Persson 2017; August and Anderson 1987); authentic vocalizations have more acoustic variability than posed vocalizations (Anikin and Lima 2017; Lavan et al. 2015), and so on. However, it must still be determined how all this potentially available acoustic information is processed. One possibility is that listeners go from acoustic features directly to a model of the speaker’s emotional state and intentions, perhaps mapping sounds to discrete emotional states (e.g., Ekman’s basic emotions, 1992) or dimensions such as valence and arousal (Briefer 2012; Russell 1980). Alternatively, the interpretation of a vocalization could be mediated by its acoustic classification: first we recognize that we hear a laugh and then decide whether this is a laugh of genuine amusement, mere social politeness, an “evil” laugh, etc. (Provine 2001).

If acoustic classification indeed mediates interpretation of vocalizations, acoustic categories should be highly salient. In several studies of non-linguistic vocalizations (Anikin and Persson 2017; Gendron et al. 2014a) participants sometimes hesitated to attribute any particular emotion to the caller, while confidently naming the sound (e.g., as a laugh or a scream). In the visual domain, a similar dissociation has been observed between naming a facial expression and interpreting its emotional significance (Boster 2005; Gendron et al. 2014b). The receiver of a communicative display, such as a vocalization or a facial expression, may thus recognize and classify the signal itself (e.g., as a laugh or a scowl) without attributing any particular emotion to the signaler. As a result, descriptions of signals in terms of their surface form (a laugh, a scowl) and in terms of their meaning (merriment, annoyance) are complementary rather than redundant. A possible theoretical interpretation is that the identification of a communicative display precedes its attribution to a particular social or emotional cause. According to this view, sometimes known as the Identification-Attribution model, these two processes may reflect a neurological division of labor between different systems (Sperduti et al. 2014; Spunt and Lieberman 2012).

There are thus some indications that affective states may not be the only, or even the most appropriate, categories for describing the repertoire of nonverbal communicative displays. In the case of non-linguistic vocalizations, there is also a natural alternative to emotional categories, namely acoustic categorization of sounds in terms of call types.

### Human Call Types

Despite its central significance in studies of animal communication, the concept of call type has no generally accepted definition. It refers to distinguishable acoustic units (“calls”) that together comprise a species’ vocal repertoire, but the exact nature and number of such units depend on whether the main interest is in production or perception, on the chosen method of classification, on the extracted acoustic variables, and so on (Fischer et al. 2016; Kershenbaum et al. 2014). Primate vocalizations are particularly challenging to categorize, because they tend to grade into each other acoustically (Marler 1976; van Hooff and Preuschoft 2003), and because they possess a high amount of within-call variability, complicating the task of identifying discrete vocalizations with objective statistical measures (Fischer et al. 2016; Wadewitz et al. 2015). Following the ethological tradition, we provisionally define call types as distinct species-typical vocalizations whose basic spectral-temporal structure is innate (not learned).

What ultimately makes vocalizations “distinct” is their unique neurological production mechanism. In practice, however, animal researchers seldom have access to this information, so they have little choice but to record a large number of vocalizations and deduce the underlying call types, normally by means of comparing the acoustic structure and typical context in which each sound occurs (Kershenbaum et al. 2014). Some distinctions that are salient to the animals themselves may be lost in the process, because the human ear or the analytic technique misses them (lumping), and some spurious distinctions may be found between what is actually a single vocalization (splitting). Likewise, it is unclear to what extent perceptual distinctions, whether they are made by the researcher or by the animal itself, correspond to call types defined by their unique production mechanism. The task of studying human vocal behavior is further complicated by the fact that species-typical vocalizations such as laughter coexist with language and semi-linguistic interjections (such as *urgh*, *ouch*, etc.). The silver lining for researchers working with human sounds is that they have more methodological options at their disposal: unlike animal subjects, human participants can be asked to label the stimuli verbally or to classify them in some other way, providing direct access to perceptual categories distinguished by the listeners.

The research on production and perception of human vocalizations reviewed in the previous section indicates that some vocalizations such as laughter are innate—that is, their acoustic form and, to some extent, meaning are predetermined by our genetic endowment. As a result, researchers are increasingly looking for the evolutionary roots of such vocalizations, usually by comparing them with the vocal repertoire of other primates (Provine 2001; Sauter et al. 2010; Scheumann et al. 2014). By definition, the unit of analysis in such phylogenetic reconstructions is an acoustic category rather than an emotion, and the two best-known examples are laughter and vocal crying.

Laughter presumably originated in mammalian social play (van Hooff and Preuschoft 2003; Provine 2001). Acoustically, this vocalization is recognizable above all by its distinct rhythm with approximately five syllables per second (Bryant and Aktipis 2014; Provine 2001). Unlike the ingressive–egressive laughter of the great apes, humans laugh with several syllables produced on a single exhalation (Provine 2001). Nevertheless, acoustic and contextual similarities are sufficiently strong to claim that laughter is a vocalization that humans share with other great apes (Ross et al. 2009) and perhaps even with rats (Panksepp 2007). Vocal crying is another human vocalization with clear evolutionary parallels. Several studies have indicated that crying in humans is related to mother-infant separation or distress calls, which are common in many mammalian species (Lingle et al. 2012; Newman 2007; Provine 2012). In contrast to laughter, crying consists of longer voiced syllables repeated at intervals approximately corresponding to respiratory cycles (Provine 2012). The sound of crying is typically tonal, with a pronounced harmonic structure, but it may also include noisy episodes (Lingle et al. 2012). This variation within the same basic acoustic template (within-call variation) is highly informative in cries of human infants (Scheiner et al. 2002) as well as animals (Lingle et al. 2012).

Laughter and vocal cry are thus two call types whose species-typical nature in humans is widely accepted and whose evolutionary origins are relatively clear. But to complete the puzzle, we have to learn what other call types, if any, the human vocal repertoire includes. Naming studies offer a powerful method for identifying perceptually salient acoustic categories and their meaning, and we utilized this technique in addition to performing acoustic analysis (Experiment 1). However, a linguistic approach is not without its pitfalls (see the Introduction to Experiment 2), and therefore we also performed a triad classification study, which allowed us to investigate the categorization of non-linguistic vocalizations without using any verbal labels (Experiment 2). It is worth reiterating that perceptual studies can only reveal the categories distinguished by listeners, which may or may not correspond to the underlying “true” call types (i.e., vocalizations with unique, genetically determined neurological production mechanisms and evolutionary histories). Clustering based on acoustic measurements is likewise not guaranteed to produce an “objective” classification, because call types may be graded and because the choice of acoustic variables affects the outcome. In this regard, human acoustic research is not very different from the studies of vocal communication in other mammals, and the same caution is needed when interpreting its results.

To the best of our knowledge, no study has systematically analyzed the repertoire of human non-linguistic vocalizations from this acoustic perspective, only the emotional states that they convey. As a result, there is little empirical data on our chosen research questions:

1. What acoustic categories do listeners distinguish in the wide variety of human non-linguistic vocalizations?
2. To what extent is this acoustic categorization language-specific?
3. How closely does acoustic categorization map onto emotional categorization?
4. What cognitive model best describes the relation between acoustic and emotional categorization of vocalizations?

### Source of Sounds

Our research questions require that we compare acoustic and emotional categorizations of non-linguistic vocalizations. In particular, we would like to learn whether these classifications are relatively independent or redundant (e.g., whether each acoustic category closely corresponds to a single emotion), and whether one of them precedes the other. This task calls for a novel approach to collecting the audio material. Vocalizations in most existing corpora are either elicited from people who are verbally instructed to portray a particular emotion, or they are induced by an experimental manipulation (Scherer 2013). For a project aiming to describe the repertoire of non-linguistic vocalizations and investigate their association with emotion, this type of material presents three problems:

1. There is evidence that listeners can distinguish between authentic and acted vocalizations (Anikin and Lima 2017; Bryant and Aktipis 2014; Gervais and Wilson 2005). This raises concerns about the latter’s ecological validity, suggesting that voluntarily produced vocalizations in some cases may deviate from the natural, spontaneous form.
2. Listeners can extract more information from vocalizations produced by members of the same cultural group, indicating that there is important cultural variation in human non-linguistic vocalizations (Elfenbein and Ambady 2002; Koeda et al. 2013; Laukka et al. 2013). This may be problematic if the research interest concerns species-specific, rather than culture-specific, vocalizations.
3. Acted vocalizations are typically elicited by providing participants with short vignettes or asking them to imagine a scenario targeting a particular emotion (Scherer 2013), and the recordings are then validated in a multiple-choice task, often preserving only a subset with the highest recognition rate. Each vocalization in the final corpus is thus designed to be a maximally transparent vehicle for the expression of a single emotional state. This excludes sounds—presumably abundant in real life—that accompany a complex, mixed emotional experience (e.g., a blend of fear, anger, and pain experienced by someone in a fight) as well as vocal expressions not typically associated with affect (e.g., grunts of physical effort or the trembling whine of a person freezing at a bus stop).

To avoid these limitations of most available corpora, the ideal source of sounds for the current project would be a large corpus of observational material, recorded in culturally diverse locations and not tied to particular emotional states. To our knowledge, no such “perfect” collection of human vocalizations exists. As a reasonable compromise, we chose to work with the observational corpus compiled from social media and validated by Anikin and Persson (2017), which contains 260 authentic vocalizations from a wide variety of contexts. It has the advantage of containing many intense and potentially hard-to-fake (Anikin and Lima 2017) vocalizations associated with acute fright, injury, genuinely funny incidents, etc. This makes it more likely that the available material extends to extreme and socially inappropriate vocalizations. Many of these sounds may be associated with mixed emotional states (Anikin and Persson 2017), making them more realistic objects for investigating the mapping between acoustic and emotional categorizations compared to actor portrayals of discrete emotions. This corpus also goes beyond the traditional range of contexts in emotion research and includes vocalizations of pain and physical effort (for a list of contexts and audio files, see Electronic Supplementary Materials).

## Experiment 1

In this cross-linguistic naming study, participants heard non-linguistic vocalizations from real-life interactions and chose one or more verbal labels to describe each sound in terms of its acoustics (e.g., a laugh, a moan, etc.) and emotion (e.g., amusement, pleasure). To our knowledge, naming studies have not been used in this manner to compare categorizations of emotional displays in different languages. The research on facial expressions (Ekman et al. 1969; Izard 1994) is different in that it focused on cross-cultural recognition of particular emotions, rather than on the categorization of facial behavior in each language. More relevant to our purpose, there is a growing body of cross-linguistic research in domains other than emotion, such as color (Berlin and Kay 1991), body parts (Enfield et al. 2006), locomotion (Malt et al. 2010), and verbs of breaking-cutting (Majid et al. 2008). The principal technique, known as the Nijmegen method (Slobin et al. 2014), is to elicit free descriptions of events or objects. The more often participants apply the same name to two stimuli, the more similar these two stimuli are assumed to be. A low-dimensional representation of these similarities together with lexical information may be referred to as a conceptual space, semantic space, or semantic map (on terminological distinctions, see Zwarts 2010). Languages are compared in terms of the overall structure of their respective semantic spaces as well as the extensions and prototypical core meanings of particular words (Zwarts 2010).

Semantic spaces may include both gradients and discontinuities. Where important natural discontinuities exist, languages are likely to make a categorical distinction. For instance, speakers of different languages agree on the exact transition point between walking and running, demonstrating a clear categorical distinction between these two modes of locomotion (Malt et al. 2010). In contrast, the distinctions are more likely to be language-specific in domains containing gradients with no abrupt discontinuities. For example, within each of the two basic gaits of walking and running, there is a continuum carved up differently by different languages (Slobin et al. 2014). Despite this general rule, continuous domains may also have natural attractors, so that categories in different languages may have the same best exemplars. For instance, while the range of hues falling under the local term for “red” varies considerably across languages, people in most societies agree on what constitutes a good example of pure red. It is therefore generally accepted that focal colors are universal, probably because of the physiology of human vision (Berlin and Kay 1991; Lindsey and Brown 2009).

By applying this linguistic method combined with acoustic analysis to non-linguistic vocalizations, we aimed to address the first two research questions, namely to identify the most salient call types distinguished by listeners and to compare this categorization in different languages. If some human vocalizations are species-typical, as is often suggested (Provine 2001; Ross et al. 2009; Sauter et al. 2010; Scheumann et al. 2014), we hypothesized that they should be recognized cross-culturally as distinct perceptual categories. The semantic spaces of sound names should thus have comparable global configurations in different languages, although the number of subdivisions within each major category and the extensions of different terms could be language-specific.

In addition to naming each sound, we also asked participants to interpret it emotionally. This allowed us to explore the mapping of call types to emotions and to address research question 3, namely to test whether: (a) there is a close correspondence between the perceived call type and the perceived emotion, or (b) acoustic and emotional categorizations are relatively independent (non-redundant).

Finally, to shed some light on the cognitive processes involved in the interpretation of non-linguistic vocalizations (research question 4), we tested whether there would be any preference to perform the acoustic and emotional categorization of vocalizations in a particular order, and whether these naming decisions would differ in speed, subjective certainty, and consistency. The Identification-Attribution model predicts that the surface form of the communicative signal—its call type—should be identified first, followed by a more elaborate interpretation in terms of the feelings and goals of the vocalizer. Alternatively, acoustic and emotional categorizations could represent two independent processes that run in parallel rather than sequentially. In this case we should not find a consistent temporal relationship or a strong correlation between the ease of categorizing a particular sound by acoustic type and by emotion.

In pilot tests we initially followed the Nijmegen method (Slobin et al. 2014) and elicited free-text descriptions of each sound. With this design, sounds are classified in an inductive manner: each participant creates their own categories for classifying the stimuli. Our participants volunteered a manageable number of sound names, but emotion names contained many synonyms, and there was a tendency to provide complex descriptions of the hypothetical context in which vocalizing took place instead of monolexemic labels (cf. Boster 2005). We strove to keep the two naming tasks compatible and therefore opted to provide participants with a list of monolexemic sound names and emotion names that were commonly used by participants in the pilot study.

By analogy with Berlin and Kay’s (1991) technique for eliciting basic color terms, we were less interested in polylexemic descriptions, very low-frequency words, terms that are mostly applicable to animal but not human sounds, and recent foreign loans. To make sure the list of sound names was comprehensible, we also checked the frequencies of all potential sound names in English, Swedish, and Russian, whether or not these words were actually used by participants in the pilot study. All high-frequency words were included in the labels (see Electronic Supplementary Materials). Eventually we chose 16 sound names in English, but in Swedish and Russian this would have required including some uncommon words, so we reduced the number of sound names to 12. The list of emotion labels in all three languages included 16 terms (see Fig. 3 for a complete list of labels for each language).

### Materials and Methods

#### Stimuli

The experimental stimuli consisted of 132 authentic non-linguistic vocalizations (63 by men, 69 by women and children), which were selected by stratified random sampling from a larger, previously validated corpus (Anikin and Persson 2017). This corpus was compiled from online videos of people engaged in a variety of emotionally charged and easily interpretable activities, such as cleaning a blocked toilet or eating exotic foods (disgust), playing with distorting web cameras or watching a friend take a spectacular tumble (amusement), lifting heavy weights (effort), and so on, for a total of nine categories: amusement, anger, disgust, effort, fear, joy, pain, pleasure, and sadness. Strictly speaking, these categories are contextual-emotional, since we only know in what context the vocalization was emitted, not the “true” affective state of the caller. The sounds were on average 2.2 ± 1.8 s in duration. The callers were primarily English speakers, but we tried to avoid language-specific emblems such as *ouch*, *yuck*, etc. For the most part, the tested sounds are thus free from any phonemic structure.

#### Participants

Participants (*N* = 64) were mono- or bilingual speakers of Swedish (*n* = 20), English (*n* = 19), or Russian (*n* = 25). The Swedish- and English-speaking participants were recruited among students and junior staff at Lund University and tested in person, ensuring that every participant rated all 132 stimuli. Russian participants were recruited and tested online, resulting in some incomplete reports (18 out of 25 Russian participants completed over 85% of trials).

#### Procedure

The experiment was performed in a web browser (See Electronic Supplementary Materials, Figure A1). Participants chose one or more suitable sound names and emotion names from a list of alternatives. This task is different from the inductive categorization in the pilot tests, since participants chose among a limited number of provided categories. They could change their minds and correct their answers as many times as needed, until they clicked the *Next* button and moved on to the next sound. It took 30–40 min to rate 132 sounds.

To assess the facility of naming acoustic types and emotions, participants could have been asked to do these two tasks sequentially, in random order. However, responses were generally slow (mean total time for both tasks 25 s), making it hard to know which processes might be responsible for differences in response times. We therefore opted to present both sound names and emotion names on the same screen, which allowed us not only to measure response times, but also to evaluate individual preferences for starting by naming either the sound or the emotion. To control for the general tendency to start with the left-hand side of the screen, for half of the participants in each language sound names were on the left-hand side, and emotion names were on the right-hand side of the screen. For the other half of participants, this order was reversed.

#### Statistical Analysis

All analyses were performed in R (R Core Team 2016).

##### Semantic Space of Sound Names

In order to construct semantic spaces representing the perceptually salient acoustic categories and dimensions along which they are distinguished, we calculated pairwise Euclidean distances between all stimuli based on how often participants chose the same name for two sounds. This was done separately for each language, after which the resulting distance matrices were averaged across languages. Distance matrices were analyzed using principal components analysis (PCA) and multi-dimensional scaling (MDS). We defined a cluster as a group of stimuli with the same most commonly chosen name. Centroids were calculated by taking a weighted mean of the coordinates of all sounds in a cluster, using as weights a product of (1) the average subjective certainty with which each sound was named by participants and (2) the proportion of the most common sound name out of all sound names applied to the same stimulus by different participants. This ensured that cluster centroids were close to the most representative sounds in each category.

We also performed affinity propagation clustering of the semantic distance matrix using *apcluster* R package (Bodenhofer et al. 2011). To find optimal clustering solutions, we varied the parameter *q* (sample quantile of the preference with which a data point becomes a centroid), which modulates the propensity of clustering algorithm for splitting or lumping. We then examined the quality of the resulting clustering solution by measuring (1) the average Silhouette Index, which is a measure of compactness and purity of clusters, and (2) the similarity of the clustering solution to the clusters defined by the most common name of each sound chosen by the participants (cf. Gamba et al. 2015).

##### Analysis of Acoustic Data

All acoustic measurements were taken from the original acoustic analysis of the corpus as reported in Anikin and Persson (2017) and Anikin and Lima (2017). They included measures (median and standard deviation) of amplitude, fundamental frequency (pitch), distribution of energy in the spectrum, harmonics-to-noise ratio, proportion of voiced frames, and several temporal measures, such as the number, spacing and regularity of syllables. We aimed to define the acoustic space that would optimally preserve the structure of the semantic space of sound names. To do this, we chose a subset of acoustic variables and their weights iteratively, trying to maximize the correlation between the acoustic distance matrix (Euclidean distances between stimuli based on a weighted linear combination of acoustic predictors) and a reference distance matrix derived from the participants’ judgments.

A subset of twelve acoustic predictors listed and explained in Table 1 proved optimal for maximizing the correlation with the semantic distance matrix (based on sound names in all three languages). In practice, the weights of acoustic variables did not have to be adjusted much to achieve optimal correlation with any of the other explored distance matrices (Table 2, first column): Cronbach’s alpha for weights optimized for different targets was 0.95; 95% CI [0.91, 0.99]. We then employed two classification algorithms to predict the chosen sound names in each language. The more easily interpreted multinomial regression was trained on the first two principal components of the acoustic matrix in order to visualize the acoustic space in each language (Fig. 2), while the more powerful Random Forest classifier, which builds and cross-validates a large “forest” of decision trees (Breiman 2001), was trained on the 12 individual predictors to estimate the extent to which objective acoustic measurements were sufficient to predict the perceived acoustic type.

##### Relation Between Call Types and Emotions

Contingency tables describing co-occurrence of sound names and emotion names were analyzed using Random Forest. This allowed us to estimate to what extent we could predict the perceived emotion knowing the chosen sound name(s) of a sound.

To compare the speed with which participants named the acoustic type and emotion of each stimulus, we recorded the delay between sound onset and (1) choosing the first sound name, (2) choosing the first emotion name, and (3) clicking the *Next* button to proceed to the next sound. Response times greater than 60 s were occasionally (~ 4% of trials) recorded among Russian participants, who took the test online without supervision. Presumably, such long delays were related to technical problems or participants taking a break, and they were removed from the analysis of response times. Time measures were log-transformed due to a right skew in their distribution and analyzed using a Gaussian model with two random effects: sound and participant. This and other linear models were fit using Markov chain Monte Carlo in the Stan computational framework (Stan Development Team 2014).

Subjective certainty in the chosen answer was indicated separately for emotion name and sound name as, “*Don’t know*”, “*Unsure*”, or “*Sure*”. It was analyzed using ordinal logistic regression, again with two random effects. The consistency of participants’ choices was operationalized as normalized entropy of all names chosen for a particular stimulus by all participants who had rated it, separately for sound names and for emotion names:

$${\text{entropy}} = - {\text{sum}}(\log_{2} ({\text{a}}/{\text{sum}}({\text{a}}))*{\text{a}}/{\text{sum}}({\text{a}}))/\log_{2} ({\text{number}}\_{\text{alternatives}})*100\% ,$$

where *a* was a vector of the same length as the number of alternative answers (number_alternatives, which was either 12 or 16) consisting of the number of times each sound name or emotion name was chosen. Because both the number of alternatives and the total number of responses per term varied, entropy was normalized to range from 0 to 100%. The distribution of entropy of 132 sounds was approximately normal, and therefore it was analyzed using Gaussian models.

The sounds, *R* scripts, raw data, additional tables and graphs can be accessed at http://cogsci.se/publications.html.

### Results

#### Perceptually and Acoustically Distinct Call Types

**Fig. 1:**
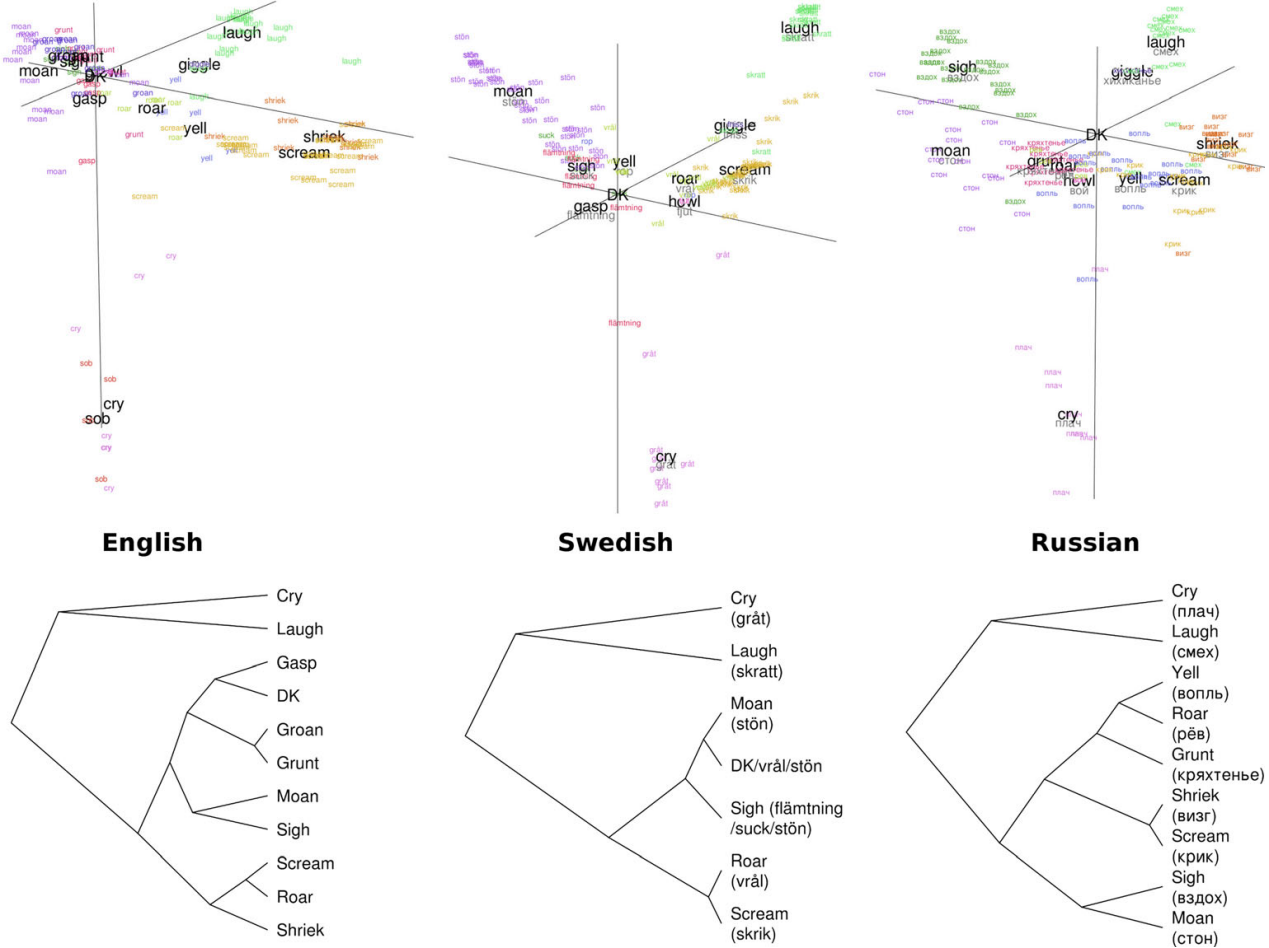
Top panel: semantic space representing naming distinctions in English, Swedish, and Russian. Text labels are positioned in prototypicality-adjusted cluster centroids. Bottom panel: cladograms of the major call types. Affinity propagation clustering with q selected manually (0.5 for English, 0.3 for Swedish, 0.45 for Russian). *DK* don’t know

In each language, we identified the most common name for each of 132 sounds and constructed a language-specific semantic space, in which the relative distance between any two stimuli depends on how often they were described with the same word. In all three languages, the first three principal components explained > 80% of variance in the resulting distance matrix, suggesting that three-dimensional solutions were adequate. Figure 1 (top panel) shows the semantic spaces of sound names for English, Swedish, and Russian. Each text label represents a single sound, labeled with its most commonly chosen name. The closer two sounds are in the graph, the more often they were given the same name by different participants. In addition, for each sound name the central location of stimuli with this name—cluster centroid—is shown in bold letters. For example, the centroids for the English words scream and shriek are close to each other, indicating that this distinction was not particularly consistent.

Based on visual inspection, semantic spaces of sound names are remarkably similar for all three languages: one dimension separates moan-like from scream-like sounds, while two more dimensions separate laughing and crying from all other sounds. More formally, the distance matrices for English, Swedish and Russian sound names are strongly correlated: *r* > 0.8 for all three pairs of languages (see Table 2).

As shown in the cladograms in the bottom panel of Fig. 1, in all three languages the most fundamental distinction was made between laughing, crying, and the remaining vocalizations. Beyond these three major groups, the order of separation between clusters was more language-specific. The languages also differed in the depth of classification: English appears to have the richest sound vocabulary with at least ten consistently labeled acoustic types, compared to as few as six in Swedish and seven or eight in Russian. The exact number is hard to determine, since measures of clustering quality indicated several valid clustering solutions. The cladograms in Fig. 1 are merely one possible interpretation of the major call types based on the naming data in these three languages.

We also performed acoustic analysis to determine how subjective categorization of non-linguistic vocalizations related to objective acoustic differences between these sounds. Since the semantic spaces of sound names were so similar in English, Swedish, and Russian, we averaged the corresponding distance matrices from all three languages and used this averaged matrix to find an acoustic space of non-linguistic vocalizations that would represent, as faithfully as possible, the acoustic distinctions observed by speakers of these languages. As shown in Table 1, a subset of 12 weighted acoustic variables maximized the correlation between acoustic and semantic distance matrices (*r* = 0.50).

**Table 1:** Variables used to construct the acoustic space and their weights optimized for maximum correlation between acoustic and semantic spaces

Variable	Interpretation	Weight	Loadings	
			PC1	PC2
Amplitude, median	Median root square amplitude (loudness)	0.61	0.13	−0.17
Proportion of voiced frames	How much of the sound is voiced	1.28	0.24	−0.41
Pitch, median	Fundamental frequency or perceived pitch (manually checked)	1.89	0.75	0.19
Pitch, SD		0.79	0.21	0.1
First quartile, median	First quartile of spectral energy distribution	1.42	0.53	0.04
First quartile, SD		0.74	0.15	0.16
Spectral entropy, SD	SD of the entropy of spectral energy distribution	0.9	0.03	0.16
Interburst interval, median	Time between vocal bursts (amplitude peaks)	0.58	0.01	−0.03
Interburst interval, SD		1.73	0.04	−0.07
Number of bursts	Total number of amplitude peaks per sound	1.61	−0.11	0.81
Syllable length, median	Length of continuous vocal segments	0.77	0	−0.16
Syllable length, SD		0.48	0.04	−0.08

**Fig. 2:**
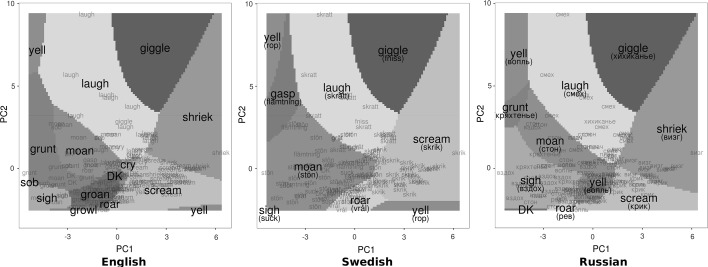
Acoustic models for classifying vocalizations based on sound names chosen by English, Swedish, and Russian participants. Shaded areas show the acoustic class predicted by a multinomial regression model using two first principal components of 12 acoustic features (see Table 1). Small labels show the position of individual stimuli and their call type

In other words, we asked the following question: What acoustic characteristics do we have to measure in order to separate the sounds into the same groups as did our participants when they named the sounds? The matrix of the chosen 12 (scaled and weighted) acoustic variables had only two strong principal components, which together explained 64% of variance. The first principal component correlated primarily with median pitch and the second with the number of vocal bursts (Table 1; Fig. 2). Based on the available acoustic measurements, it appears that participants distinguished between call types primarily based on their pitch, the number and irregularity of syllables, the balance between voiced and unvoiced parts, and some spectral characteristics.

It is also interesting to determine to what extent the classification of sounds into call types can be reproduced using objective acoustic measurements. Adjusted Rand Index demonstrates a much higher agreement of the actual naming with a clustering solution based on distances in the averaged semantic space (0.46, 0.48, and 0.49 for English, Swedish, and Russian, respectively) than with a clustering solution based on distances in the acoustic space (0.14, 0.15, and 0.13). The reason is that acoustically the stimuli are highly graded. As can be seen in the scatterplot in Fig. 2, sounds form a single cloud, with no clear clusters and a lot of overlap between call types. This contrasts with the relatively well-separated clusters in Fig. 1. Using the 12 acoustic variables listed in Table 1, a Random Forest classifier correctly predicted the chosen sound name approximately 40% of the time in English, 62% in Swedish, and 54% in Russian. We also repeated Random Forest classification after pooling sound names into six major categories (laugh, cry, scream, moan, sigh, and roar) plus one residual unclassified “other” category. With these seven categories, classification accuracy was approximately 60% for all three languages.

Our findings thus indicate that participants classified sounds into call types more consistently than could be expected given the available acoustic measures. This result should be treated with some caution, however, since several call types were represented by only a few sounds (e.g., gasp, howl, etc.). The most common types, such as laughs and screams, also had high recognition rates in Random Forest models (75% and better), suggesting that classification accuracy by acoustic models might improve with a larger training sample.

#### How Do Call Types Map onto Emotion?

**Fig. 3:**
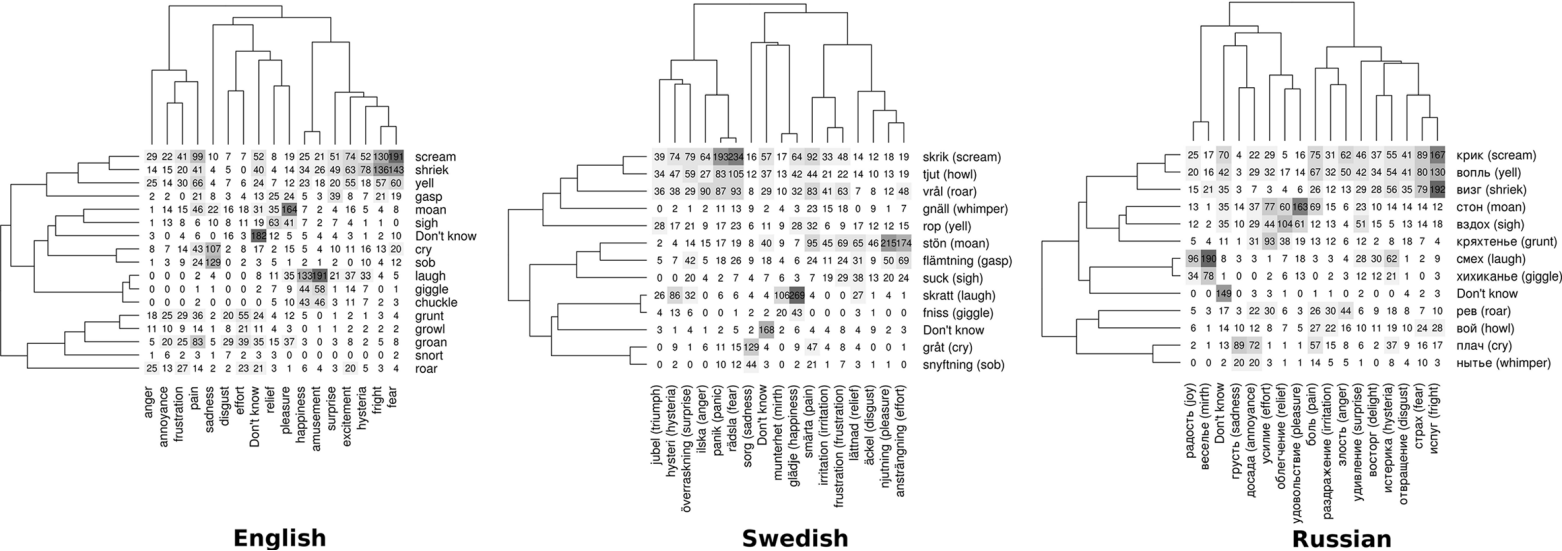
Co-occurrence of sound names (vertical) and emotion names (horizontal) in English, Swedish, and Russian. Each cell shows the number of times a participant chose a particular sound name and emotion name to describe the same sound. The labels are ordered by hierarchical clustering, so that similar terms are placed close together, resulting in the dendrograms shown next to each table

To explore the correspondence between naming the sound and naming the speaker’s emotion, we analyzed contingency tables of sound and emotion names (Fig. 3). For example, the cell in the top left corner for English shows that a sound was simultaneously labeled *scream* and *anger* in 29 individual trials, whereas the combination of *scream* and *fear* was more common (191 trials). A Chi square test performed on this table proved that these acoustic-emotional classifications were not independent (English: *χ* ^2^ = 8568, *df* = 256; Swedish: *χ* ^2^ = 7761, *df* = 192; Russian: *χ* ^2^ = 7102, *df* = 192; *p* < 10^−15^ for all three). However, the association between naming the acoustic type of a vocalization (e.g., a scream) and naming its emotion (e.g., fear) was far from perfect. Based on the chosen sound name, a Random Forest classifier correctly predicted the chosen emotion name approximately 60% of the time in English, 50% in Swedish, and 60% in Russian. Knowing what speakers called a sound thus provided roughly half the information needed to predict its perceived emotion.

Of course, perfect correspondence is less likely when there are more emotion names than sound names, as was the case in Swedish and Russian. However, the association between call type and emotion was similarly imperfect in English, which had 16 sound names and 16 emotion names. Furthermore, the lack of one-to-one mapping is not only due to the presence of close synonyms among the available verbal labels. For example, when a participant classified a sound as a scream, the perceived emotion varied widely and included quite distinct contexts, such as fear, pain, delight, surprise, etc. Moans, grunts, and sighs also varied considerably in their emotional interpretation. In contrast, laughing and crying were more closely associated by participants with a particular emotional state (amusement/joy and sadness, respectively; see Fig. 3).

#### Ease and Consistency of Naming the Sound Versus Naming the Emotion

Participants started by naming the emotion in ~ 3% of trials when emotion names were on the right, but they started by naming the sound in ~ 27% of trials when sound names were on the right: odds ratio = 28, 95% CI [7, 141]. If the relative position of sound names and emotion names on the screen was the only factor affecting the order of responses, the probability of answering left-to-right should have been the same regardless of whether sound names or emotion names were on the left. Instead, we observed a bias to name the sound before naming the emotion.

Median time needed to name both the sound and its emotion was 14 s, and median time needed to choose the first of these names was 5 s. Controlling for the order in which the two blocks were presented on the screen, it took 850 ms [740, 960] longer to choose the first emotion name versus the first sound name. This observation confirms that participants preferred to name the sound before naming its emotion.

**Fig. 4:**
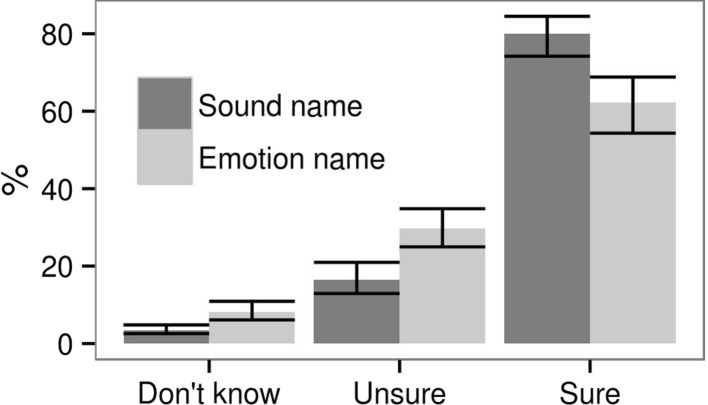
The probability of expressing different levels of certainty in the chosen sound names and emotion names for all three language groups combined. Median of the posterior distribution and 95% CI

Subjective certainty in the given answers, measured on a scale of 1–3 (*Don’t know*—*Unsure*—*Sure*), was on average 2.76 [2.75, 2.78] for sound names and 2.54 [2.52, 2.56] for emotion names. The proportion of “*Sure*” ratings was higher for sound names, while the proportions of “*Don’t know*” and “*Unsure*” ratings were higher for emotion names (Fig. 4). The results were similar for all three languages (not shown). Furthermore, there were 7.4% of trials in which participants named the sound but not the emotion, whereas the reverse pattern of naming the emotion but not the sound occurred in only 1.7% of trials: odds ratio = 5.2 [4.2, 6.5]. Participants thus named the sound with more certainty than they named the emotion.

Normalized entropy was considerably lower for sound names than for emotion names (49 vs. 59%, difference = 9.6% [7.7, 11.6], Cohen’s *d* = 0.76). Low entropy means that participants mostly agreed on what to call a particular stimulus, whereas high entropy means that different participants chose many different terms for the same stimulus. Our results thus suggest that sound names were chosen more consistently than emotion names. In Swedish and Russian, this could be due to the smaller number of available alternatives: 12 sound names versus 16 emotion names. However, we corrected for the number of alternatives and used normalized entropy. Moreover, in English there were equal numbers of sound names and emotion names (16 of each), but the entropy of sound names was still 7.3% [5.0, 9.5%] lower.

We expected that it would be easier to name the call type than to name the emotion only for those sounds that were the most distinct acoustically (e.g., laughs), while for other sounds it would be easier to name the emotion than to name the call type. However, the average certainty in the given sound names was higher than the average certainty in the given emotion names for all 40 (12 + 12 + 16) sound names in the three languages and for all but one emotion (disgust). Sound names were also chosen faster than emotion names for all call types in all languages except *flämtning* (gasp) in Swedish, *snort* in English, and *pёв* (roar) in Russian. In addition, there was a strong positive correlation between the speed of naming each sound and naming its emotion: *r* = 0.75, 95% CI [0.62, 0.85]. Similarly, the certainty in the choice of sound name (averaged per sound) correlated with the certainty in the choice of emotion name: *r* = 0.75, 95% CI [0.63, 0.86]. There was also a positive correlation between the entropy of sound names and emotion names: *r* = 0.59 (95% CI [0.44, 0.72]). The speed, certainty and consistency of naming a particular sound were thus strongly correlated with the speed, certainty and consistency of naming the emotion that it expressed.

To summarize, English-, Swedish-, and Russian-speaking participants in Experiment 1 demonstrated a high level of agreement when classifying non-linguistic vocalizations into approximately six major call types, which could also be defined in terms of objectively measured acoustic features. More fine-grained classification into acoustic subtypes was generally less consistent both across and within languages. The classifications of a sound in terms of its acoustic type and emotion were neither totally independent nor redundant: some call types were strongly associated with a single emotion, while others were perceived to express a variety of states. It seemed more natural to name the sound before naming its emotion, apparently for most call types and emotions. However, these two processes were not independent: sounds that were easy to classify acoustically were also easy to interpret in terms of the caller’s emotion, while acoustically unnameable sounds remained emotionally opaque.

## Experiment 2

Cross-linguistic naming studies, such as the one above, have their limitations. One problem is that the availability of verbal labels in a language is not a prerequisite for distinguishing categories of stimuli. For example, Yucatec Maya does not possess two separate words for disgust and anger, but there is evidence that speakers still perceive the corresponding facial expressions as two distinct categories (Sauter et al. 2011). Often modifiers allow speakers to make subtle distinctions despite a paucity of basic lexemes (Malt et al. 2010). In other cases the abundance of language-specific lexical distinctions may exaggerate the apparent complexity and culture-specificity of a cognitive domain and obfuscate its underlying universality. For example, similarities between household utensils based on direct non-linguistic comparisons are more consistent across languages compared to similarities derived from verbal labeling of such objects (Ameel et al. 2005; Malt et al. 1999).

In other words, the presence or absence of a linguistic distinction in several languages can be suggestive, but in itself it can neither prove nor falsify the universality of the corresponding conceptual distinction. It is therefore desirable to obtain language-independent evidence. Moreover, in Experiment 1 participants were forced to choose among 12 or 16 pre-given labels, further restricting the possible patterns of classification. Given these limitations, we also tested the same 132 sounds in another experiment, avoiding verbal labels altogether and aiming to obtain an estimate of “naked” perceived similarity between stimuli.

To do this, we used the triad classification task, which is an established tool for studying the categorization of multidimensional stimuli (Alvarado 1996; Raijmakers et al. 2004). Participants in a triad task are presented with three stimuli at a time and select two that are the most similar. These decisions can be used to estimate the perceived “distances” between stimuli. Since in Experiment 1 we discovered that call types were highly salient to listeners, we hypothesized that this distance matrix would be more compatible with the distance matrix calculated in Experiment 1 based on the chosen sound names, rather than with the distance matrix based on emotion names. Participants’ choices in a triad task depend on the instructions: they have to be told on what basis they are supposed to compare the stimuli in each triad. We loaded the dice against the hypothesis and specifically asked participants to choose based on the similarity of underlying emotional states, not the similarity of acoustic characteristics.

The triad task has previously been applied to the classification of emotional vocalizations: Green and Cliff (1975) tested 11 sounds, one for each emotion. However, it is impossible to discover an alternative clustering with so few stimuli. Besides, Green and Cliff worked with artificial and speech-like material (recited letters of the alphabet) rather than natural vocalizations. Our study is thus the first to use the triad task for label-free classification of human vocalizations.

### Materials and Methods

#### Stimuli

We used the same 132 sounds as in Experiment 1.

#### Participants

Participants in the triad task were recruited on the campus of Lund University or online, through advertisements and personal contacts. All participants who performed at least ten out of forty-two trials were included in the analysis (*N* = 241). The experiment was available in three languages: Swedish (*n* = 156 participants), English (*n* = 77) and Russian (*n* = 8). Since the Russian sample was too small to construct a distance matrix, we only present the results for the Swedish and English samples.

#### Procedure

The experiment was written in html/javascript and made available online. Participants performed the test in common rooms at the university or at home. It took approximately 10–15 min to complete the entire test (132 sounds in 42 triads), although incomplete tests were also accepted. All data was completely anonymous and the online test could be interrupted at any time.

A standard version of the triad classification task (Raijmakers et al. 2004) was used. Participants were presented with three sounds at a time, and they could replay each sound as many times as they needed before indicating which two sounds in the triad were emotionally more similar. Just like Nijmegen method of free-text labeling in the pilot version of Experiment 1, categorization in the triad task is inductive, in the sense that the nature of categories is not predetermined and their number is not limited. The instructions, visible throughout the experiment, specifically asked to choose based on the emotional state of the caller. Each of 132 sounds was presented once in random order.

#### Statistical Analysis

The output of the triad task was analyzed using a Bayesian model. The model assumes that each sound is embedded in a *d*-dimensional space and that for every triad the participant’s choice is a function of the relative distances between the three sounds. The pair of sounds with the smallest distance is the one most likely to be chosen by the participant. To find the posterior distribution of the embedding in *d*-dimensional space, the model was fit using Markov chain Monte Carlo in the Stan computational framework (Stan Development Team 2014).

**Fig. 5:**
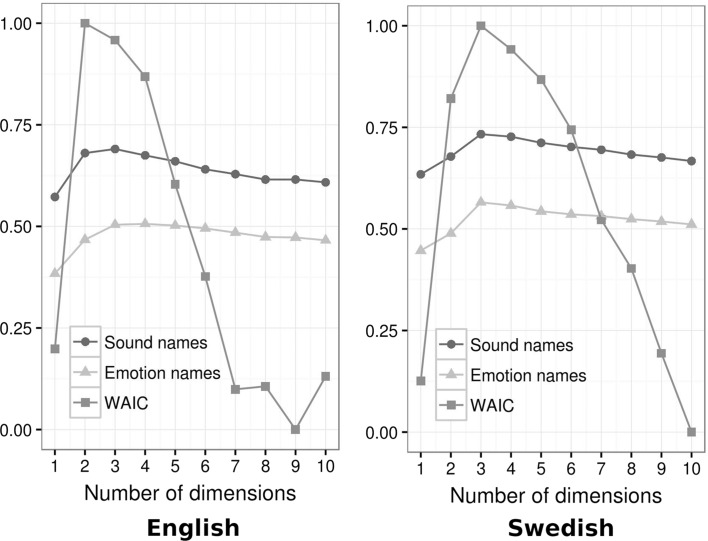
Model fit as a function of its dimensionality for the triad classification task. Shown: Pearson’s correlation with distance matrices from Experiment 1 based on naming the sound or emotion and normalized negative WAIC (larger is better)

Since dimensionality was hard-coded in the generative model, we explored models with different numbers of dimensions and estimated how well each described the actual responses of participants. Watanabe-Akaike Information Criterion (WAIC), which is an approximation to leave-one-out cross-validation, was used as a measure of overall fit (Watanabe 2010). In addition, we calculated the correlation between the distance matrices based on linguistic labeling in Experiment 1 (either sound names or emotion names, averaged across three languages) and the distance matrix in Experiment 2 estimated by a generative model with *d* dimensions, separately for Swedish and English (Fig. 5).

### Results and Discussion

The first step was to determine how many dimensions were necessary to represent the configuration of stimuli corresponding to the distinctions made by participants in the triad task. A three-dimensional model achieved optimal correlation with the distance matrices from Experiment 1 based on naming both the sound and its emotion for both English and Swedish data (Fig. 5). WAIC suggested that three dimensions were optimal for Swedish and two or three for English; we therefore focused on three-dimensional models.

**Fig. 6:**
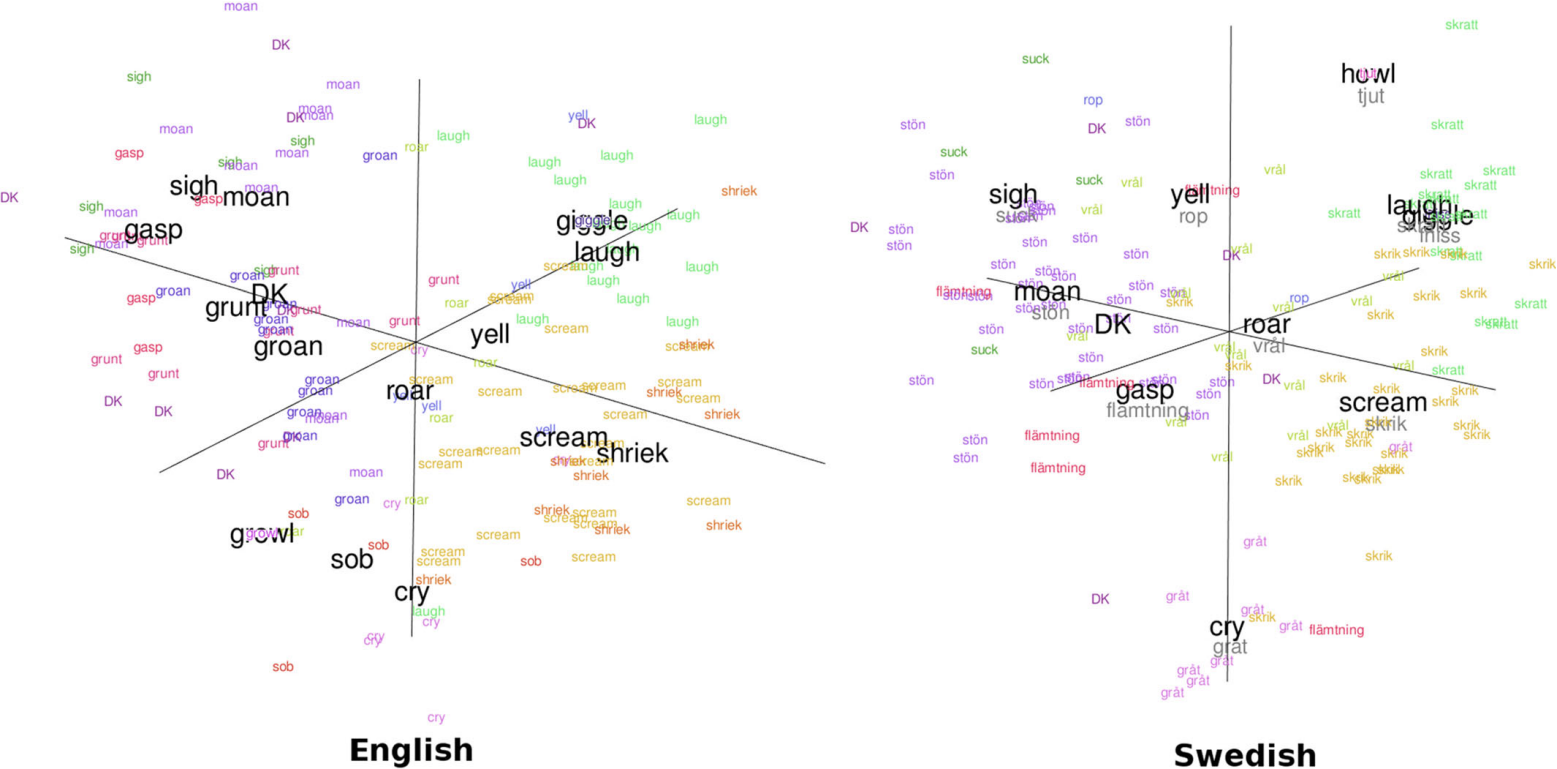
Three-dimensional configuration of stimuli in the triad task. Each sound is labeled and color-coded by its most common name in Experiment 1 (cf. Fig. 1). Prototypicality-adjusted centroids are shown in large bold font. Origin lies in the center of gravity of each cloud. *DK* don’t know

For both English and Swedish, the distance matrix from the triad task was more similar to the distance matrix from Experiment 1 based on sound names (*r* = 0.69 for English and 0.73 for Swedish data) than to the distance matrix from Experiment 1 based on emotion names (*r* = 0.50 and 0.57, respectively; Table 2). A visual inspection of the configuration of stimuli that best represented similarity judgments made by participants in the triad task (Fig. 6) confirmed that this configuration was qualitatively similar to the semantic space of sound names in Fig. 1. Once again, laughs and cries formed clearly separated clusters, while the remaining sounds were spread out in a cloud from sighs and moans to screams. The main difference between this configuration and semantic spaces in Experiment 1 was that the clusters were less compact in the triad task. The reason may be that participants in Experiment 1 had to choose among a few available verbal labels, whereas similarity judgments in the triad task were unrestrained, allowing more subtle distinctions.

**Table 2:** Pearson’s correlations between the distance matrices in Experiments 1 and 2

	Acoustic analysis	Sound names EN	Sound names SV	Sound names RU	Sound names EN + SV + RU	Emotion names EN	Emotion names SV	Emotion names RU	Emotion names EN + SV + RU	Triad classif. EN	Triad classif. SV
Sound names EN	0.47										
Sound names SV	0.46	0.85									
Sound names RU	0.48	0.8	0.8								
*Sound names EN* + *SV* + *RU*	**0.5**	0.94	0.95	0.92							
Emotion names EN	0.34	0.68	0.65	0.62	0.69						
Emotion names SV	0.33	0.65	0.64	0.58	0.67	0.87					
Emotion names RU	0.43	0.69	0.68	0.73	0.74	0.8	0.77				
*Emotion names EN* + *SV* + *RU*	**0.38**	0.72	0.7	0.68	**0.75**	0.95	0.95	0.91			
Triad classif. EN	0.5	0.62	0.68	0.63	0.69	0.45	0.46	0.52	0.5		
Triad classif. SV	0.48	0.67	0.71	0.67	0.73	0.52	0.52	0.55	0.57	0.75	
*Triad classif. EN* + *SV*	**0.52**	0.69	0.74	0.69	**0.75**	0.51	0.51	0.57	**0.56**	0.96	0.91

Bold values indicate the most relevant information

Acoustic analysis = distance matrix based on 12 acoustic variables with weights optimized for maximum correlation with each target distance matrix (see Figure S2), thus producing the highest achievable correlation

Sound names EN/SV/RU: distance matrix based on English/Swedish/Russian sound names

Sound names EN + SV + RU: averaged distance matrix for sound names in all three languages

Emotion names EN/SV/RU: distance matrix based on English/Swedish/Russian emotion names

Emotion names EN + SV + RU: averaged distance matrix for emotion names in all three languages

Triad classif. EN/SV: distance matrix based on the triad classification task in the English/Swedish sample

Since so few Russian-speaking participants took part in the triad classification task, no comparison could be made for this language. Moreover, the English group in the online-based triad task is not guaranteed to consist entirely of native speakers (in contrast to Experiment 1, where participants were tested in person and were native speakers), potentially limiting the compatibility of English data from the two studies. Despite these limitations, Experiment 2 has demonstrated that, overriding the explicit instructions to choose based on emotion, participants made similarity judgments that were more compatible with verbal classification of stimuli into acoustic categories than into emotional categories. This convergent evidence highlights the perceptual salience of acoustic categories and confirms that linguistic labeling in Experiment 1 provided valid information about the underlying cognitive representation of non-linguistic vocalizations.

## General Discussion

To investigate the relation between acoustic categories (e.g., laughter or moan) and perceived emotion, we analyzed acoustically 132 sounds from a corpus of authentic non-linguistic vocalizations (Anikin and Persson 2017) and compared their verbal classification by acoustic category and emotion by native speakers of English, Swedish, and Russian (Experiment 1). We found strong parallels across all three languages in the distinguished acoustic categories, which was further confirmed using a nonverbal classification test (Experiment 2). In line with acoustic research in other primates (Fischer et al. 2016), human vocalizations appear to be highly graded, and all sounds apart from laughing and crying can be roughly aligned along a single dimension, which acoustically corresponds to pitch. Based on the analyzed sample of sounds and languages, the conceptual space of non-linguistic vocalizations thus appears to be three-dimensional, and the most salient acoustic categories are: laughing, crying, screaming, and moaning. We suggest that these categories may correspond to species-typical call types—innate vocalizations that are produced and recognized in all cultures. Roaring and sighing are two more candidate call types, but the evidence in their case is less conclusive.

This list of perceptually distinct vocalizations can only be regarded as preliminary, since it critically depends on the range of tested sounds. For example, there were no completely voiceless sounds in the corpus, likely influencing the apparent semantic extension of the word *sigh*. Moreover, we only examined three languages from the Indo-European family, and the variation in sound-related vocabulary may become larger if more distantly related languages are compared. However, even with only three languages, it is already clear that the number of “basic” (i.e., perceptually and acoustically distinct) call types is considerably smaller than the number of available lexemes for acoustic categories in the general vocabulary.

To understand why this is so, it is helpful to distinguish between extension and connotation of sound names. For example, the words for breathy sounds in English, Swedish, and Russian appear to differ primarily in their extensions: only English has a consistent ingressive–egressive distinction at the level of basic lexemes (*sigh* versus *gasp*), the Russian *вздox* (sigh) apparently allows for relatively more voicing, etc. There are also sound names that differ primarily in their connotations, such as *groan*/*moan* in English or *rop*/*vrål* in Swedish. These words were often applied to the same sound by different participants, depending on which emotion they perceived. They are therefore not fully synonymous, but it may still be unwarranted to claim that they represent two different vocalizations, since these semantic distinctions are neither acoustically robust nor consistent across languages. Finally, words like *laughing*/*giggling*/*chuckling*, *crying*/*sobbing*, and *screaming*/*shrieking*/*yelling*, as well as the equivalent terms in Swedish and Russian, are close synonyms that overlap in both extension and connotation in all three languages. In such cases, the likely interpretation is that these words refer to subtypes of what is perceptually a single vocalization.

As a result, we are left with only a handful of cross-culturally recognized and acoustically definable call types, perhaps as few as four to six. This relatively small number may come as a surprise, considering the larger number of lexemes for non-linguistic vocalizations and of emotions that can be correctly detected based on vocal cues (9–16 emotions in Cordaro et al. 2016; 14 emotions in Simon-Thomas et al. 2009). The number of call types we identified also falls far short of that ascribed to the great apes. Estimates vary, but gorillas may have about 16 call types (Fossey 1972), bonobos 12–19 (Bermejo and Omedes 2000; de Waal 1988), chimpanzees 13–24 (Goodall 1986; Marler 1976), and orangutans 32 (Hardus et al. 2009). An intriguing possibility is that these estimates of the size of vocal repertoire in apes are inflated, because within-call acoustic variation is easily mistaken for distinct call types. For example, by simply varying the amount of nonlinearities such as subharmonics and deterministic chaos, a nearly tonal vocalization can be made bark-like and almost unrecognizable as an instance of the same call (Fitch et al. 2002). Once the production mechanism of each call is better understood, some ape vocalizations may thus be reclassified as variations of the same basic type.

The relatively small number of identified human call types does not contradict the well-established fact that a rich variety of affective states can be recognized from non-linguistic vocalizations. Even a few distinct vocalizations may still be sufficient for expressing a wide range of meanings, provided that within-type acoustic variation is meaningful (Scheiner et al. 2002; Wadewitz et al. 2015). For instance, the exact manner of laughing may tell the listener as much as the fact that this is a laugh rather than, say, a grunt. Consistent with this explanation, the distinction between tonal and noisy sounds did not appear to contribute to the categorization of sounds by call type in this study, whereas this acoustic parameter is of major importance for the categorization of the same sounds by emotion (Anikin and Persson 2017). Relatively tonal and noisy vocalizations of the same basic acoustic type may thus be associated with different emotions. The expressive range of each call type may be further enhanced by contextual information and integration of sound with input from other sensory modalities. For example, visible tears make crying less ambiguous and enhance the impression of sadness (Provine 2012; Provine et al. 2009).

It is also quite possible that humans possess more call types than we have identified, but these vocalizations lack monolexemic labels, at least in the investigated Indo-European languages. These call types may also fail to be consistently distinguished by participants and acoustic models, perhaps because the boundaries between them are blurred. In fact, our acoustic analysis revealed that most call types were highly graded, complicating their clustering based on the extracted acoustic features and limiting the accuracy with which acoustic models could predict the sound name in each language. A possible objection is that the acoustic characteristics we measured do not describe the sounds comprehensively. However, even fewer acoustic variables sufficed for machine learning algorithms to achieve accuracy on a par with human raters when classifying the original corpus by emotion (Anikin and Persson 2017). A more serious limitation of the current research is that our sample of 132 sounds may not be large enough or comprehensive enough to be considered representative of the range of non-linguistic vocalizations people produce. Our analysis needs to be extended, using a larger and more diverse collection of vocalizations, ideally recorded from an even broader range of contexts and from several cultural groups.

The interpretation we favor is that humans do possess species-typical vocalizations, but these are graded and further masked by the great variety of culturally learned non-linguistic vocalizations. Only the most salient and involuntary vocalizations remain universal and distinct enough to be perceived categorically in all cultures, with laughter being the paradigmatic example. We did not test for categorical perception per se, but the compact clustering of laughing and crying in the naming task, and particularly in the triad classification task, strongly suggests that at least these two vocalizations are perceived as qualitatively different from all other sounds, which is in line with previous studies of these two vocalizations (Lingle et al. 2012; Provine 2012). The fact that the separation between acoustic types made by participants was more consistent than might be expected based on acoustic measurements also implies their categorical perception, which can be verified in future studies. Ultimately, it would be also be illuminating to analyze the neurological and physiological processes involved in the production of each call type putatively identified in perceptual studies. This would both verify the validity of suggested acoustic categories and determine whether their universality is due to innateness or some other processes driving cross-cultural convergence.

In addition to identifying the major call types and their meaning, it is important to specify a cognitive model of the relation between sound and emotion classification by the listener. As a step in this direction, we compared the two tasks—naming the sound and naming its emotion—in terms of decision time, preferred order, subjective certainty, and consistency. Naming a sound acoustically (as a laugh, a scream, etc.) was associated with faster responses, greater certainty and higher consistency across participants compared to naming its emotion (Experiment 1). Intriguingly, this was the case for practically all analyzed vocalizations, not only for some particular classes. Furthermore, asked to compare the sounds based on the underlying affective state of the caller, participants still appeared to think largely in terms of acoustic categories (Experiment 2). At the same time, there was a close relation between the ease of acoustic and emotional interpretations. If a sound could not be named, its emotion could not be determined, and vice versa: sounds that were easy to name were also more easily and consistently interpreted in terms of the underlying emotion.

A parsimonious explanation for these observations is that every vocalization is initially categorized acoustically and then interpreted in terms of the caller’s emotion or intention, in accordance with the identification-attribution model (Spunt and Lieberman 2012). This task is streamlined when the vocalization belongs to a common and acoustically well-defined category, such as laughing or screaming. This would explain the strong correlation between the ease of naming the sound and the ease of naming its emotion: the identification of a particular call type carries useful information for the receiver, since each call type is associated with only a restricted range of emotions. Nevertheless, the association between call type and emotion is not redundant; instead, it turns out to be considerably more complex than might have been expected.

This calls for a complementary approach to the study of non-linguistic vocalizations—one mindful of acoustic types as such, rather than solely their potential for expressing emotion. We hope that this approach may provide a more comprehensive and phylogenetically informed account of vocal behavior, shedding new light on human nonverbal communication and bringing it more in line with research on vocal communication in other animals.

## Electronic supplementary material

Below is the link to the electronic supplementary material.

Supplementary material 1 (DOCX 237 kb)
