# Supplementary material for: Human Non-linguistic Vocal Repertoire: Call Types and Their Meaning
Source: J Nonverbal Behav. 2017 Sep 30;42(1):53–80. doi: 10.1007/s10919-017-0267-y (PMC5816134; doi:10.1007/s10919-017-0267-y)
Supplement: Supplementary file 1 — Supplementary material 1 (DOCX 237 kb) [file 10919_2017_267_MOESM1_ESM.docx]

Appendix

# Supplementary materials

# Note: audio files and raw data are available from <http://cogsci.se/personal/results.html>

**Figure A1.** The English version of the naming experiment, with sound names on the left and emotion names on the right. For half of the participants, this order was reversed to test the preferred order of sound-emotion classification.


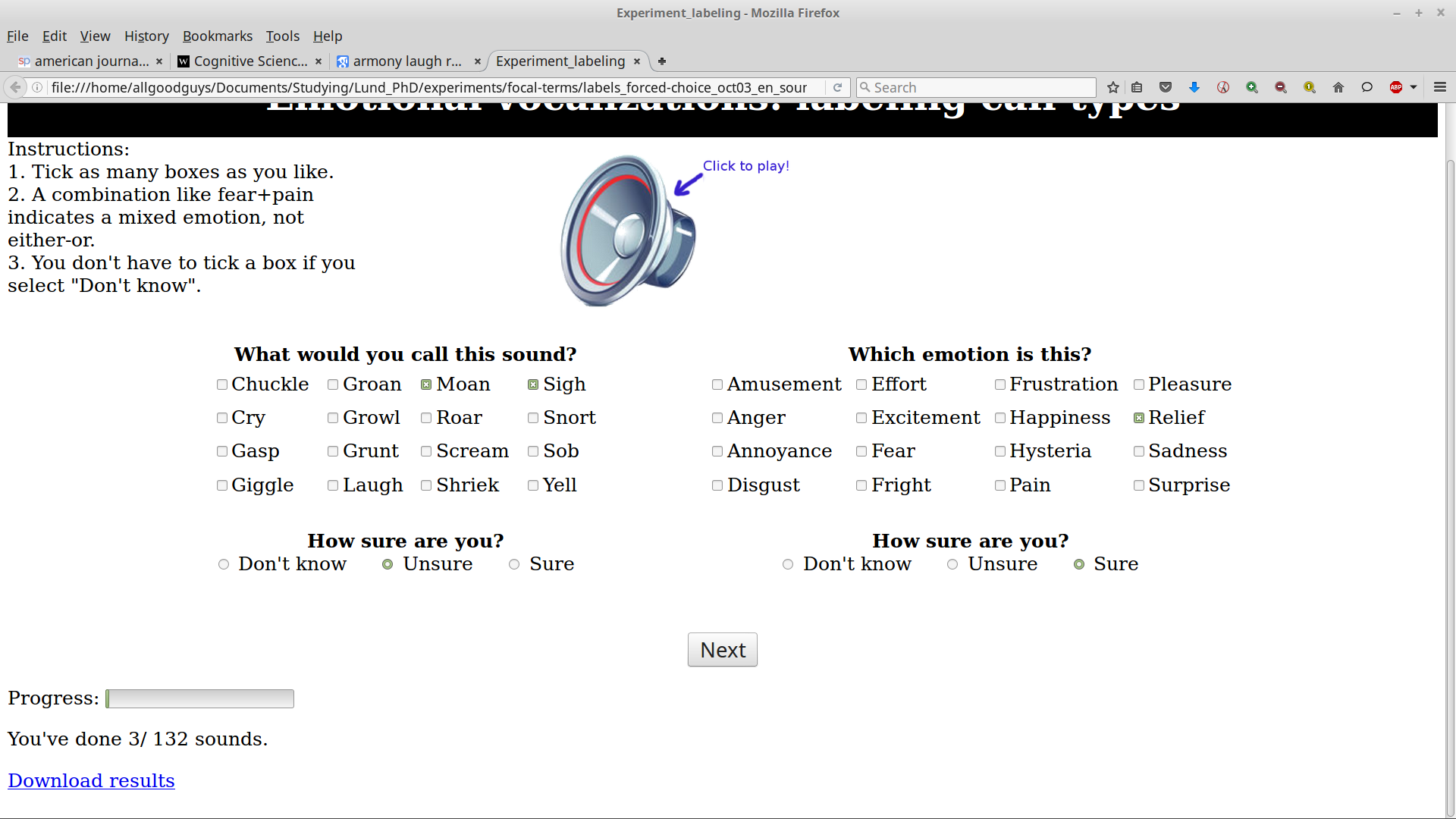

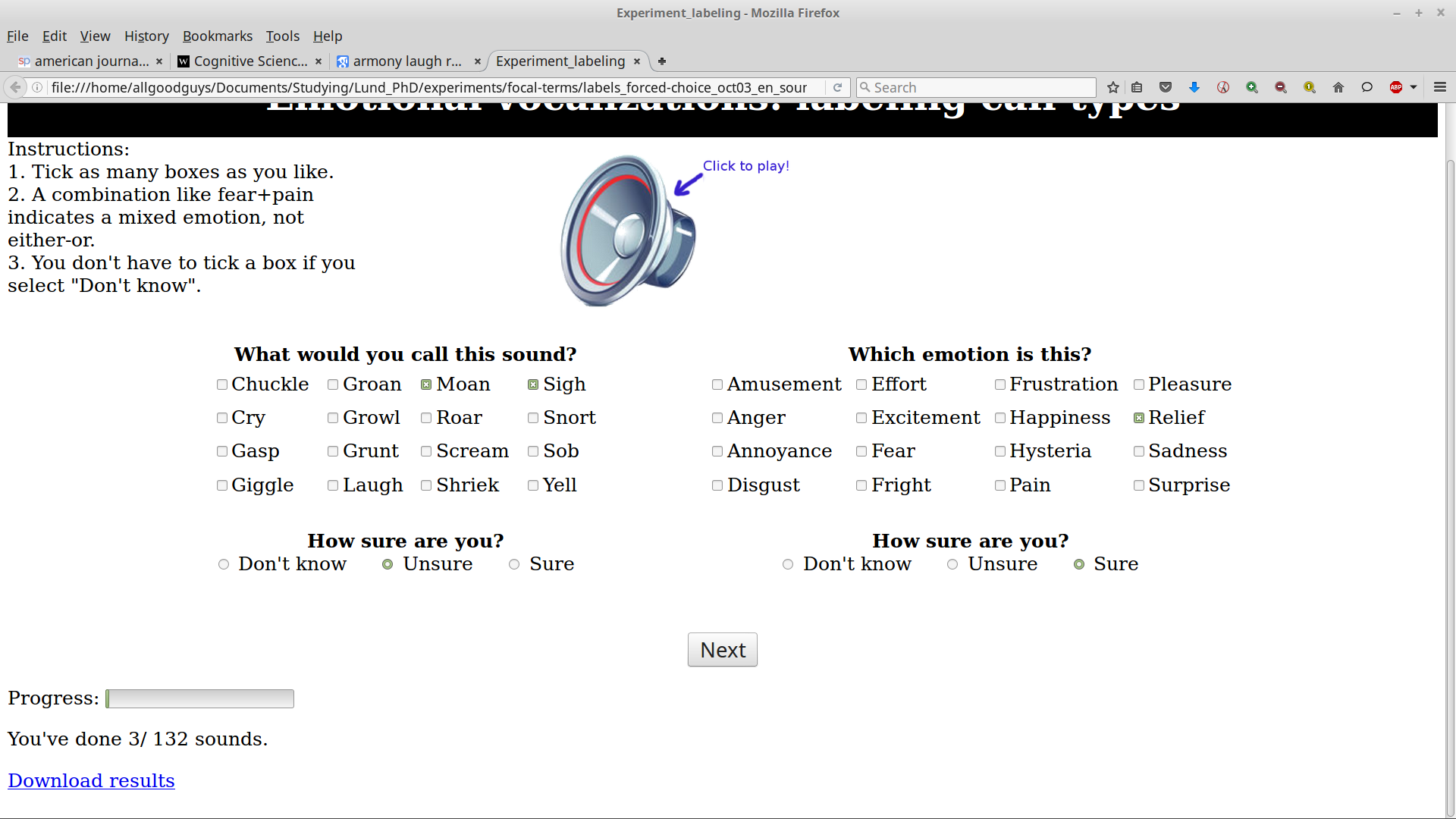


| **Table A1.** Names of sounds and emotions in English, Swedish and Russian: consistency and clustering | | | | | | | | | | |
| --- | --- | --- | --- | --- | --- | --- | --- | --- | --- | --- |
| Sound name  (English equivalent) | No. of sounds | Median distance, %^a^ | Cluster purity, %^b^ | Median entropy, %^c^ |  | Emotion name  (English equivalent) | No. of sounds | Median distance, %^a^ | Cluster purity, %^b^ | Median entropy, %^c^ |
| Chuckle | 0 | - | - | - |  | Amusement | 17 | 32 | 100 | 48 |
| Cry | 7 | 34 | 64 | 29 |  | Anger | 5 | 43 | 83 | 73 |
| Gasp | 4 | 25 | 100 | 43 |  | Annoyance | 1 | - | - | 74 |
| Giggle | 1 | - | - | 38 |  | Disgust | 5 | 36 | 100 | 46 |
| Groan | 11 | 41 | 85 | 61 |  | Effort | 6 | 33 | 86 | 62 |
| Growl | 1 | - | - | 65 |  | Excitement | 10 | 57 | 91 | 69 |
| Grunt | 11 | 45 | 73 | 56 |  | Fear | 16 | 51 | 67 | 59 |
| Laugh | 17 | 36 | 100 | 41 |  | Fright | 6 | 41 | 86 | 61 |
| Moan | 14 | 38 | 93 | 52 |  | Frustration | 1 | - | - | 60 |
| Roar | 6 | 36 | 100 | 64 |  | Happiness | 0 | - | - | - |
| Scream | 26 | 42 | 63 | 47 |  | Hysteria | 2 | 56 | 100 | 75 |
| Shriek | 10 | 35 | 38 | 42 |  | Pain | 15 | 55 | 83 | 61 |
| Sigh | 6 | 30 | 100 | 43 |  | Pleasure | 13 | 49 | 93 | 53 |
| Snort | 0 | - | - | - |  | Relief | 5 | 41 | 100 | 59 |
| Sob | 4 | 24 | 100 | 42 |  | Sadness | 10 | 36 | 100 | 35 |
| Yell | 5 | 33 | 56 | 50 |  | Surprise | 3 | 51 | 100 | 73 |
| “Don't know” | 9 | 43 | 100 | 51 |  | “Don't know” | 17 | 55 | 94 | 53 |
| **TOTAL^d^** | **132** | **37** | **79** | **48** |  | **TOTAL^d^** | **132** | **45** | **89** | **56** |
|  |  |  |  |  |  |  |  |  |  |  |
| Flämtning (gasp) | 6 | 54 | 43 | 53 |  | Ansträngning (effort) | 13 | 52 | 93 | 58 |
| Fniss (chuckle) | 1 | - | - | 31 |  | Frustration | 4 | 47 | 67 | 55 |
| Gnäll (whimper) | 0 | - | - | - |  | Glädje (happiness) | 22 | 71 | 85 | 48 |
| Gråt (crying) | 9 | 38 | 100 | 37 |  | Hysteri (hysteria) | 2 | 41 | 100 | 78 |
| Rop (shout) | 2 | 47 | 67 | 61 |  | Ilska (anger) | 7 | 42 | 78 | 68 |
| Skratt (laugh) | 17 | 37 | 100 | 27 |  | irritation | 0 | - | - | - |
| Skrik (scream) | 30 | 41 | 100 | 48 |  | Jubel (triumph) | 4 | 41 | 100 | 69 |
| Snyftning (sob) | 0 | - | - | - |  | Lättnad (relief) | 6 | 45 | 100 | 62 |
| Stön (moan) | 40 | 56 | 93 | 52 |  | Munterhet (mirth) | 0 | - | - | - |
| Suck (sigh) | 4 | 39 | 100 | 55 |  | Njutning (pleasure) | 14 | 53 | 100 | 57 |
| Tjut (yell, shriek) | 1 | - | - | 36 |  | Panik (panic, fright) | 5 | 48 | 33 | 58 |
| Vrål (roar) | 17 | 40 | 85 | 54 |  | Rädsla (fear) | 15 | 51 | 62 | 61 |
| “Don't know” | 5 | 50 | 45 | 51 |  | Smärta (pain) | 12 | 54 | 86 | 66 |
| **-** | - | - | - | - |  | Sorg (sadness) | 9 | 36 | 100 | 37 |
| - | - | - | - | - |  | Äckel (disgust) | 4 | 39 | 100 | 43 |
| - | - | - | - | - |  | Överraskning (surprise) | 5 | 47 | 71 | 65 |
| - | - | - | - | - |  | “Don't know” | 10 | 49 | 34 | 53 |
| **TOTAL^d^** | **132** | **45** | **89** | **47** |  | **TOTAL^d^** | **132** | **52** | **80** | **57** |
|  |  |  |  |  |  |  |  |  |  |  |
| Вздох (sigh) | 20 | 42 | 87 | 47 |  | Боль (pain) | 11 | 70 | 39 | 75 |
| Визг (shriek, screech) | 17 | 36 | 81 | 48 |  | Веселье (mirth) | 18 | 59 | 100 | 60 |
| Вой (howl) | 1 | - | - | 66 |  | Восторг (delight) | 2 | 35 | 100 | 71 |
| Вопль (yell) | 19 | 45 | 76 | 68 |  | Грусть (sadness) | 6 | 43 | 86 | 55 |
| Крик (shout, scream) | 17 | 55 | 61 | 54 |  | Досада (annoyance) | 6 | 49 | 29 | 73 |
| Кряхтенье (grunt) | 8 | 46 | 100 | 62 |  | Злость (anger) | 6 | 47 | 86 | 73 |
| Нытье (whimper) | 0 | - | - | - |  | Испуг (fright) | 24 | 55 | 96 | 62 |
| Плач (crying) | 9 | 32 | 100 | 47 |  | Истерика (hysteria) | 2 | 36 | 100 | 76 |
| Рёв (roar) | 4 | 37 | 100 | 64 |  | Облегчение (relief) | 7 | 49 | 100 | 61 |
| Смех (laugh) | 17 | 37 | 100 | 42 |  | Отвращение (disgust) | 1 | - | - | 60 |
| Стон (moan) | 17 | 48 | 94 | 52 |  | Радость (joy) | 1 | - | - | 82 |
| Хихиканье (chuckle) | 1 | - | - | 29 |  | Раздражение (irritation) | 1 | - | - | 73 |
| “Don't know” | 2 | 45 | 100 | 63 |  | Страх (fear) | 2 | 36 | 100 | 69 |
| **-** | - | - | - | - |  | Удивление (surprise) | 7 | 67 | 58 | 73 |
| - | - | - | - | - |  | Удовольствие (pleasure) | 13 | 58 | 100 | 60 |
| - | - | - | - | - |  | Усилие (effort) | 11 | 54 | 85 | 57 |
| - | - | - | - | - |  | “Don't know” | 14 | 51 | 78 | 64 |
| **TOTAL^d^** | **132** | **42** | **85** | **53** |  | **TOTAL^d^** | **132** | **54** | **82** | **64** |
| ^a^ Normalized median distance between sounds in a cluster and its centroid. Smaller distances indicate more compact clusters. ^b^ A measure of separation between clusters (the proportion of sounds belonging to a cluster within its bounding sphere). The closer it is to 100%, the more complete the separation.  ^c^ Median entropy of all sounds in the cluster. Low entropy indicates that only a few labels were applied consistently to all sounds in the cluster.  ^d^ Weighted means of cluster distances, purity or entropy. | | | | | | | | | | |

**Table A2.** The contexts in which sounds in each category were emitted

| Emotion | Contexts | Qualitative acoustic description |
| --- | --- | --- |
| Amusement | Pranks, failed stunts, distorting web camera, social play | Laughs |
| Anger | Malfunctioning computer, losing a game or detecting a cheater, tantrum | Roars or noisy screams, growls |
| Disgust | Unblocking a clogged toilet, food challenges (surströmming, baby food) | Grunting, retching noises, 'Aah', 'Ugh' |
| Effort | Weightlifting, amateur gymnastics (pull-ups, push-ups) | Grunts, roars |
| Fear | Scare pranks, bungee-jumping, “haunted house” attraction, spiders | Screams |
| Joy | Opening exam results, “We're pregnant!” videos, sport fans cheering after a score | Screams, laughs, roars, sighs |
| Pain | Men: failed stunts, sport injuries; women: giving birth | Roars, screams, moans |
| Pleasure | Having sex or masturbating (usually without a video track, so that the authenticity of these vocalizations cannot be guaranteed) | Moans, grunts |
| Sadness | Complaining and crying about someone's death, broken relationship, a sad movie, etc | Crying with tears |

**Table A3.** The frequency of different sound names in English, Swedish, and Russian

| **ENGLISH** | | | | |
| --- | --- | --- | --- | --- |
|  | [**http://www.wordandphrase.info/frequencyList.asp**](http://www.wordandphrase.info/frequencyList.asp)  **(noun, rank)** | [**http://www.wordandphrase.info/frequencyList.asp**](http://www.wordandphrase.info/frequencyList.asp)  **(verb, rank)** | [**http://www.wordcount.org/main.php**](http://www.wordcount.org/main.php)  **(noun or verb, rank)** | [**https://books.google.com/ngrams/**](https://books.google.com/ngrams/)  **(noun or verb, freq%)** |
| Laugh(ter) | 3618 | 862 | 2610 | .0017 |
| Chuckle | 11178 | 6419 | 18371 | .000014 |
| Giggle | 10391 | 7473 | 16269 | .0001 |
| Snicker | 20668 | 19937 | 49128 (snigger) | .000016 |
| Guffaw | 26794 | 28249 | 55322 | .000012 |
| Cackle | 27431 | 20043 | 37122 | .000018 |
| Cry | 3465 | 1355 | 2955 | .002 |
| Sob | 11538 | 7348 | 18033 | .00012 |
| Whimper | 20498 | 14497 | 30700 | .00005 |
| Wail | 13739 | 9068 | 22423 | .00011 |
| Whine | 14102 | 8055 | 21747 | .0001 |
| Howl | 11987 | 8535 | 19540 | .00013 |
| Weep | 24253 | 5380 | 13454 | .00025 |
| Blubber | 25815 | 31224 | 44651 | .00003 |
| Scream | 5300 | 2130 | 6860 | .0005 |
| Shout | 7536 | 2167 | 5392 | .00048 |
| Yell | 13762 | 2644 | 18106 | .00024 |
| Squeal | 17103 | 12036 | 25980 | .00005 |
| Shriek | 12882 | 9509 | 21498 | .00009 |
| Screech | 14478 | 12252 | 23939 | .00005 |
| Yelp | 18383 | 17033 | 42059 | .00003 |
| Roar | 7069 | 5333 | 9408 | .0003 |
| Bellow | 13760 | 12640 | 24234 | .00004 |
| Snarl | 16693 | 12649 | 26666 | .00005 |
| Growl | 12679 | 8917 | 23995 | .0001 |
| Rumble | 12274 | 8913 | 18911 | .0001 |
| Grunt | 9842 | 9901 | 19689 | .0001 |
| Moan | 12320 | 7783 | 14165 | .00013 |
| Groan | 11912 | 7293 | 14565 | .00016 |
| Snort | 17187 | 8437 | 22400 | .00006 |
| Sigh | 5943 | 3371 | 6386 | .0006 |
| Wheeze | 25429 | 14959 | 34241 | .000027 |
| Gasp | 10329 | 5730 | 12922 | .00017 |
| Pant | 2706 | 9974 | 40769 | .00005 |
| Whoop | 24010 | 18514 | 32776 | .000035 |

| **RUSSIAN** | | | |
| --- | --- | --- | --- |
|  | [**http://www.ruscorpora.ru/search-main.html**](http://www.ruscorpora.ru/search-main.html) **(#entries)** | [**http://www.artint.ru/projects/frqlist.php**](http://www.artint.ru/projects/frqlist.php) **(downloaded; freq)** | [**https://books.google.com/ngrams/**](https://books.google.com/ngrams/)  **(noun or verb, freq%)** |
| Смех / смеяться | 9037 / 4608 | 95.12 / 166.68 | .0013 / .0005 |
| Хихиканье / хихикать | 111 / 132 | 1.35 / 10.77 | .0000007 / .00001 |
| Гогот / гоготать | 122 / 22 | 4.16 / 4.16 | .000017 / .000004 |
| Фырканье / фыркать | 166 / 79 | 1.29 / 6.92 | .000008 / .000005 |
| Плач / плакать | 2154 / 5290 | 27.73 / 120.71 | .00046 / .0007 |
| Вой / выть | 2064 / 593 | 24.85 / 17.51 | .00076 / .0001 |
| Вопль / вопить | 1856 / 290 | 26.69 / 15.98 | .0002 / .00004 |
| Рыдание / рыдать | 300 / 488 | 8.26 / 18.30 | .000036 / .00008 |
| Всхлипывание / всхлипывать | 52 / 178 | 1.9 / 12.06 | .000006 / .000015 |
| Нытье / ныть | 142 / 277 | 1.78 | .000028 / .00003 |
| Причитание / причитать | 43 / 165 | 1.84 / 5.51 | .000018 / .00003 |
| Скулеж / скулить | 41 / 120 | 1.16 / 4.84 | .000004 / .00001 |
| Стенание / стенать | 66 / 48 | 1.16 / 1.22 | .00001 / .00001 |
| Писк / пищать | 604 / 117 | 5.75 / 4.28 | .00008 / .00001 |
| Хныканье / хныкать | 28 / 159 | - / 2.20 | .000004 / .000016 |
| Крик / кричать | 8271 / 4719 | 104.85 / 220.36 | .0012 / .00058 |
| Вскрик / вскрикнуть | 239 / 164 | 5.14 / 20.57 | .00003 / .000018 |
| Вопль / вопить | 1856 / 290 | 26.69 / 15.98 | .00025 / .00004 |
| Визг / визжать | 1505 / 231 | 23.75 / 16.04 | .00015 / .00002 |
| Визжание | 15 | - | .000001 |
| Рев / реветь | 2226 / 430 | 30.61 / 26.99 | .0004 / .00004 |
| Рычание / рычать | 219 / 139 | 4.96 / 14.14 | .000028 / .00002 |
| Рычанье | 51 | - | .000004 |
| Рыкание / рыкать | 13 / 5 | - | .000001 / .000001 |
| Рыканье | 18 | - | .000001 |
| Кряхтенье / кряхтеть | 57 / 55 | 1.22 / 13.53 | .000007 / .000004 |
| Хмыканье / хмыкать | 14 / 9 | 2.26 / 2.26 | - / .000001 |
| Хрюканье / хрюкать | 76 / 34 | - / 1.16 | .0000002 / .000004 |
| Сопение / сопеть | 153 / 93 | 3.43 / 12.92 | .000016 / .000007 |
| Сипение / сипеть | 17 / 21 | - / 3.73 | .0000015 / .000001 |
| Стон / стонать | 1796 / 322 | 21.79 / 26.93 | .0003 / .000048 |
| Вздох / вздыхать / вздохнуть | 1698 / 323 / 740 | 25.28 / 35.44 / 141.64 | .0002 / .000046 / .0001 |

| **SWEDISH** | | | |
| --- | --- | --- | --- |
| **Noun** | **svensk-frekvensordlista_100000.txt (rank)** | **Verb** | **svensk-frekvensordlista_100000.txt (rank)** |
| Skratt | 1732 | skratta | 2363 |
| Fnitter | 38520 | fnittra | 60207 |
| Fniss | 5020 | fnissa | 29109 |
| Flabb | 92116 | flabba | 81159 |
| Garv | 24660 | garva | 19960 |
| Kackel | 62813 | kackla | - |
| Gråt | 8955 | gråta | 2517 |
| Gnäll | 6981 | gnälla | 6333 |
| Snyft | 11381 | snyfta | 52084 |
| Snyftning | - |  |  |
| Böl | - | böla | 32342 |
| Gny | 67079 |  |  |
| Yl | - | yla | 40921 |
| Klagan | 30158 | klaga | 2630 (non-specific) |
| Skrik | 7782 | skrika | 3669 |
| Rop | 15040 | ropa | 6995 |
| Skri | 62424 |  |  |
| Skrän | - | skräna | - |
| Vrål | 29674 | vråla | 25680 |
| Tjut | 36141 | tjuta | 39738 |
| Skrål | - | skråla | 52280 |
| Brummande | 80797 | brumma | 75775 |
| Morrande | 56319 | morra | 43879 |
| Surr | 20641 | surra | 25021 |
| Grymtande | - | grymta | - |
| Fnys | 47465 | fnysa | 58113 |
| Knotande | - | knota | - |
| Knorrande | - | knorra | - |
| Stön | 21439 | stöna | 52955 |
| Jämmer | 71628 | jämra (sig) | - |
| Flämtande / flämtning | 41962 / - | flämta | - |
| Suck | 1550 | sucka | 25655 |
| Rosslande | - | rossla | - |
| Kräkning | - | kräkas | 10323 |
| Prat | 5907 | prata (non-specific) | 512 |
